# Contraceptive uptake and associated factors among women in the immediate postpartum period at Kawempe Hospital

**DOI:** 10.1186/s12905-022-01856-1

**Published:** 2022-07-07

**Authors:** Noor Nakiwunga, Othman Kakaire, Cynthia Kuteesa Ndikuno, Rita Nakalega, Nelson Mukiza, Susan Atuhairwe

**Affiliations:** 1 Department of Obstetrics and Gynecology, Makerere University College of Health Sciences, P.O Box 7072, Kampala, Uganda; 2 Rinecynth Advisory, Kampala, Uganda; 3 Makerere University-Johns Hopkins University (MU-JHU) Research Collaboration, Kampala, Uganda; 4 Baylor College of Medicine Children’s Foundation, Kampala, Uganda

**Keywords:** Contraceptive uptake, Immediate postpartum period, Family planning, Women

## Abstract

**Introduction:**

Within Africa, contraceptive use is low although about 214 million women who are not using contraception want to avoid pregnancy. In Uganda, modern contraceptive uptake is at 35% resulting in unwanted or unplanned pregnancies which may increase morbidity and mortality among children and mothers. Contraceptive uptake at 6 weeks postpartum is encouraged but it is not very effective since there is low attendance during this visit. Additionally, some women may have become sexually active by the visit at 6 weeks postpartum leading to early conception.

**Objectives:**

This study sought to determine contraceptive uptake in the immediate postpartum period and the associated factors among women delivering at Kawempe Hospital.

**Methods:**

This study employed a cross-sectional study design where 397 women aged 18–49 years were recruited using systematic random sampling. The women who were discharged within 72 h after delivery were considered. Data collection was done using an interviewer-administered data collection tool. Data was double entered into EpiData version 4.2 and analyzed using STATA version 13 at univariate using descriptive statistics then at bivariate and multivariate levels using logistic regression with contraceptive uptake as the outcome.

**Results:**

We enrolled 397 participants. Their mean age range was 18–45 years and a median of 25 years (IQR 22, 30). The majority of the participants, 333 (83.88%), were married and 177 (44.58%) were housewives or unemployed. Contraceptive uptake in the immediate postpartum period among these participants was 15.4% (61/397). The factors independently associated with immediate postpartum contraceptive uptake were grand multiparity (aOR = 2.57; 95% CI 1.11–5.95; *p* = 0.028), cesarean delivery (aOR = 2.63; 95% CI 1.24–5.57; *p* = 0.011), and prior contraceptive counseling during Antenatal (aOR = 9.05; 95% CI 2.65–30.93; *p* =  < 0.001).

**Conclusion:**

There was a 15.4% contraceptive uptake among immediate postpartum women which is very low. The factors independently associated with immediate postpartum contraceptive uptake were grand multiparity, cesarean section, and prior contraceptive counseling during antenatal care. Efforts need to be made to improve contraceptive uptake among immediate postpartum mothers such that the high unmet need for contraception is reduced and short inter-pregnancy intervals are controlled.

## Introduction

The use of modern contraceptives in Africa continues to be low at 33% despite global trends which show increasing usage, especially in North America at 75% [1]. Among those who are not using contraception in developing countries, about 214 million women want to avoid pregnancy [2]. In Uganda, 35% of postpartum women actively take up contraception [3]. Postpartum women are at a high risk of rapid repeat, unplanned pregnancy which is associated with adverse outcomes for the mother and child [4]. Therefore, it is recommended that women receive information on family planning and the health and social benefits of birth spacing during ANC, immediately after birth, and during postpartum periods [5].

Immediate post-partum contraception can be initiated within 48 h post-delivery or anytime afterward up to 6 weeks [5]. According to the report by WHO on medical eligibility criteria for contraceptive use, the recommended methods for women in the immediate postpartum period include Progestin-only pills, condoms, implants, Intrauterine devices (IUD), and Bilateral Tubal ligation (BTL) [6]. About 67% of married women in Uganda have a demand for family planning and 58% of this is being met by the modern contraceptive methods [3]. However, there is still a 28% unmet need for family planning services. It is estimated that immediate post-partum contraception can eliminate birth intervals of less than two years and avert one million out of the eleven million deaths in children less than 5 years [7].

Women are recommended to obtain a contraceptive method at the 6-week post-partum visit however, postpartum clinic attendance is poor due to several factors including transportation costs, child care, or employment obligations [8]. Even when the women return for the visits, they may have already resumed sexual activity and thus conceived before the follow-up visit. Offering modern contraceptive services as part of childbirth care increases postpartum contraceptive uptake and is likely to reduce both unintended pregnancies and pregnancies that are too closely spaced [7, 9].

The uptake of contraception within the first 72 h after delivery is advantageous since the mother is certain that she is not pregnant and it is also a good practice to increase contraceptive access in the immediate postpartum [10]. Despite this, there is still a high unmet need for contraception, especially among the immediate postpartum women (UBOS and ICF, 2017). There is also limited information concerning the uptake of contraception in the immediate postpartum period. Therefore this study aims to determine the immediate postpartum contraceptive uptake and the factors associated among women delivering at Kawempe Hospital. This will provide grounds for health workers to improve contraception service delivery to mothers in the hospital by providing grounds for reinforcement of contraceptive counseling. The results will also inform the Ministry of health sector of government on the creation of policies regarding immediate postpartum contraception.

## Methods

### Study design and site

This was a cross-sectional study done to determine the contraceptive uptake among 397 women aged 18–49 years that were in the immediate postpartum period at Kawempe hospital. The study was conducted in Kawempe hospital which is a national referral hospital in Uganda. This hospital was selected since it serves a big population of mothers from varying socio-cultural backgrounds. Approximately 50–60 deliveries are conducted per day. Mothers often stay in the hospital for 24 h after a vaginal delivery, whereas cesarean section mothers are discharged on their 3rd post-operative day. Approximately 60 mothers are discharged per day.

The contraceptive methods available in Kawempe Hospital include; permanent methods like bilateral tubal ligation (BTL) and vasectomy, Long Acting Reversible Contraceptives (LARC) like Intrauterine devices (both copper and levonorgestrel containing IUDs), and sub-dermal implants like Implanon and Jadelle, Short acting methods like combined and progestin-only oral pills and injectables. Other methods offered include barrier methods like male and female condoms plus fertility awareness-based methods like moon beads. The services offered are free of charge in the hospital.

The immediate postpartum period was defined as the period within 72 h of delivery. This time aids in the reduction of missed opportunities to initiate contraception given that most women miss 6-week postpartum visits.

### Sampling procedure

The 397 participants were selected using systematic random sampling. An average of 60 women were delivered per day in Kawempe hospital thus the estimated population of women during the study period (2 months) was 3600 and the sampling interval was 9. The research assistants (RAs) went to the postnatal ward every day and picked the register for the list of mothers who were being discharged after delivery. The first subject each day was selected randomly among the first 9 women. Thereafter, every 9th woman was enrolled based on the eligibility criteria. In case the kth participant did not give consent to participate, the next participant in line (k + 1) was considered since this was straight line sampling.

### Study variables

The independent variables were sociodemographic factors (age, marital status, educational level, monthly income, occupation, religion, spousal influence, mother’s attitude); Partner/spouse sociodemographic factors (age, religion, educational level, monthly income, occupation); Fertility and reproductive characteristics (parity, history of miscarriage, number of children, birth interval, ANC visits, type of delivery, plan to breastfeed, pregnancy intention); contraceptive knowledge (methods of contraception, history of contraceptive use); Health system factors (Contraceptive counseling during the ANC period). The dependent variable was contraceptive uptake among women discharged within 72 h post-delivery. For those who had taken up a contraceptive method, the particularly preferred method of contraception was also recorded.

### Data collection

Approval to do the study was sought from the School of Medicine Research and Ethics Committee (SOMREC) and Kawempe Hospital. Permission to carry out the study in the postnatal ward was obtained from the ward in charge. During data collection, the Principal Investigator (PI) was assisted by two trained Research Assistants (RA) who were midwives in Kawempe hospital. Each day during the study period, the PI and RAs assisted by the nurse on duty in the postnatal ward identified women who were being discharged and were within 72 h post-delivery. The participants were selected using systematic random sampling and those who gave informed consent to participate were recruited into the study. Interviews were then conducted in a place on the ward with assured privacy and confidentiality. The data was collected using interviewer administered questionnaire which was structured and pretested to capture data on both the dependent variable and the independent variables. The tool was translated to Luganda for those who did not understand English. The administration of the questionnaire took approximately 30 min per participant. The data on contraceptive uptake was confirmed from the patients’ files.

### Data management

The PI checked the questionnaires to ensure completeness and accuracy then data was double entered into EpiData version 4.2. The dataset was then exported to Stata version 13 where logistic regression was used for analysis. Variables were considered as significantly associated factors if they had a *p* value less than 0.05.

## Results

**Fig. 1:**
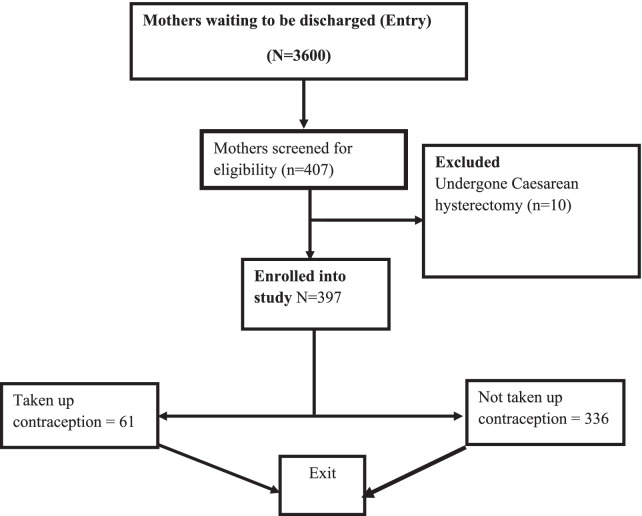
The study flow diagram. This figure is found in Additional file 1 and shows the recruitment process of study participants. There were 3600 mothers that delivered during the study period. Using systematic sampling, 407 participants were selected and screened for eligibility. Out of the 407, 10 had undergone cesarean hysterectomies and thus were excluded from the study. Therefore, 397 participants were enrolled in the study, and out of these, 61 opted to take up a contraceptive method

There was an overall total population of 3600 mothers that delivered during the study period in Kawempe Hospital. Of these 407 were screened for eligibility with 10 excluded since they had undergone a Caesarean hysterectomy (Fig. 1). Therefore, there were a total of 397 participants recruited with ages ranging from 18 and 45 years with a mean age of 26.4 years (SD = 6.0) and a median of 25 years (IQR: 22, 30). The majority of the women, 333 (83.9%), were married and 177 (44.6%) women were full-time housewives and unemployed. The details of the demographic characteristics are presented in Table 1.

**Table 1:** Demographic characteristics of women in the immediate postpartum period at Kawempe Hospital

Variable	Frequency (N = 397)	Percentage (%)
Age		
18–24 years	189	47.6
25–34 years	159	40.1
35 years and above	49	12.3
Religion		
Catholic	109	27.5
Moslem	103	25.9
Pentecostal	88	22.2
Protestant	85	21.4
Seventh Day Adventist (SDA)	12	3.0
Marital status		
Single	56	14.1
Married	333	83.9
Divorced/separated	5	1.3
Widow	3	0.8
Education level		
None	4	1.0
Primary	120	30.2
Secondary	221	55.7
Tertiary	52	13.1
Occupation		
Housewife/unemployed	177	44.6
Non professional	161	40.6
Professional	59	14.9
Household monthly income		
10,000/= to 200,000/=	165	42.3
200,001/= to 500,000/=	143	36.7
Above 500,000/=	82	21.0

The participants’ spouses/partners were aged between 19 and 64 years with a mean age of 32.1 (SD = 7.8) and a median of 30 (IQR: 26.5, 36). A majority of 314 (79.1%) of the men were involved in the family planning decision-making. The details of the spouse/partner characteristics are shown in Table 2.

**Table 2:** Characteristics of the spouses/partners of the women in the immediate postpartum period at Kawempe Hospital

Variable	Frequency (N = 397)	Percentage (%)
Age of spouse/partner		
19–24 years	50	12.6
25–34 years	220	55.4
35 years and above	127	32.0
Religion of spouse/partner		
Catholic	137	34.6
Moslem	114	28.8
Protestant	81	20.5
Pentecostal	59	14.9
Seventh Day Adventist	6	1.5
Education level of spouse/partner		
None	13	3.3
Primary	75	18.9
Secondary	227	57.2
Tertiary	71	17.9
Not sure	11	2.8
Occupation of spouse/partner		
Unemployed	13	3.3
Non-professional	296	74.6
Professional	84	21.2
Not sure	4	1.0
Is spouse/partner involved in decision making about FP		
No	73	18.4
Yes	314	79.1
Missing	10	2.5

Regarding the obstetric characteristics, the majority of the women, 203 (51.1%) were multiparous and 307 (77.3%) were planning to have their next pregnancy more than two years after the current birth. About 239 (60.2%) women had used a form of contraception before with injectable depot medroxy progesterone being the most common method used. All the women except one had plans of breastfeeding their babies. The details of the characteristics are shown in Table 3.

**Table 3:** Obstetric characteristics of women in the immediate postpartum period at Kawempe Hospital

Variable	Frequency (N = 397)	Percentage (%)
Parity		
Primipara (1)	146	36.8
Multipara (2–4)	203	51.1
Grandmultipara (5 and above)	48	12.1
Ever had an abortion		
No	294	74.1
Yes	103	25.9
Number of living children		
One child	160	40.3
Two to three children	153	38.5
Four children and above	84	21.2
Ever used any form of contraception		
No	158	39.8
Yes	239	60.2
Method of contraception used before		
Injectable contraception (DMPA)	147	61.8
Progestin only pill	34	14.3
Implants	34	14.3
IUD	16	6.7
Condoms	8	3.3
Number of ANC visits attended (n = 396)		
< = 3 visits	149	37.6
> = 4 visits	247	62.4
Received contraceptive counseling in ANC		
No	168	42.3
Yes	229	57.7
Mode of delivery		
Vaginal delivery	227	57.2
Caesarean delivery	170	42.8
Birth interval (n = 271)		
< = 24 months	82	30.3
25–48 months	102	37.6
> 48 months	87	32.1
Planned time for next pregnancy		
Never	71	17.9
Within one year	5	1.3
Within 2 years	14	3.5
More than 2 years	307	77.3
Plan to breastfeed		
Yes	396	99.7
No	1	0.3

### Prevalence of contraceptive uptake

**Fig. 2:**
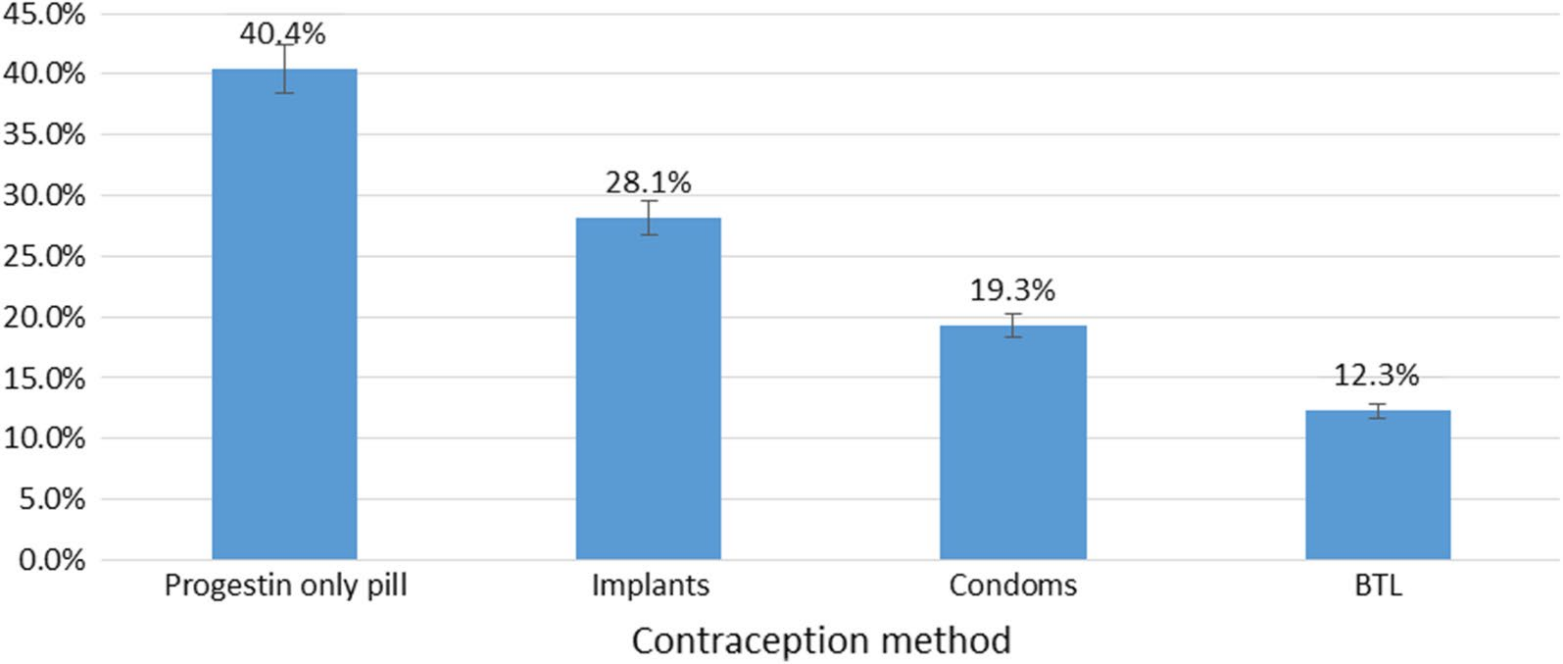
The methods of contraception selected by women in their immediate postpartum period at Kawempe Hospital. This figure is found in Additional File 2 and is a bar graph showing the proportions of women out of the 61 women that selected various methods of contraception. The methods selected include progestin-only pills, implants, condoms, and bilateral tubal ligation. Progestin-only pills were the most commonly selected method with 40.4% of the women selecting it

The prevalence of contraceptive uptake was 15.4% (61/397) (95% CI 12.1–19.3%) among the women in the immediate postpartum period. Among the 61 women with contraceptive uptake, various methods were chosen and 40.4% (95% CI 28.2–53.9%) selected the progestin-only pill (Fig. 2). The other methods selected include implants, BTL, and condoms. The least selected method at 12.3% (95% CI 5.8–24.0%) was BTL among (the women in the immediate postpartum period.

The women gave reasons as to why they chose to take up immediate postpartum contraception. Among the 336 women that opted not to take up the contraception, 321 gave reasons as to why they made this decision (Table 4). The most common reason was the women were not informed that there was an option for one to receive contraception the immediate postpartum. About 97 (30.2%) of women wanted to get the contraception after the postpartum period due to various factors like the need for time to heal, the need to see their period plus the discomfort and pain faced during the immediate postpartum period. There were a proportion of women whose decision to take up any form of contraception depended on the husband and would only consider the options after discussing with their spouses. Concerning bad attitude towards contraception, several women feared the side effects of the contraception while some have traditional beliefs that are against the use of contraception. About 8 of the women were opting for alternative methods of birth control including the use of natural family planning methods and use of herbs or traditional medicine.

**Table 4:** The reasons why women opted not to take up any form of contraception in the immediate postpartum period

Reason	Frequency	Percentage (%)
Not informed about the availability of contraception in the immediate postpartum period	113	35.20
Would like to get the contraception after the immediate postpartum	97	30.22
Not interested in using any form of contraception	32	9.97
Decision depends on the spouse	21	6.54
Bad attitude towards contraception	17	5.30
Not decided on what method to use	10	3.12
Spouse is not around	10	3.12
Opting for alternative methods of preventing pregnancy	8	2.49
Wants to conceive immediately	6	1.87
Contraceptive method not available	4	1.25
Religion does not allow use of contraceptive methods	3	1.56

Out of the 61 women who took up contraceptives, 25 gave reasons as to why they opted for immediate postpartum contraceptives (Table 5). The most common reason was that they found ease in using contraception obtained in the immediate postpartum period since they did not have to worry about any unplanned pregnancies as well as worry about their spouses refusing the use of contraception. About three women chose to use contraception to avoid transmission of HIV to their spouses thus infection control.

**Table 5:** The reasons why women had contraceptive uptake in the immediate postpartum period

Reason	Frequency	Percentage (%)
Found ease in using contraception obtained during postpartum period	12	48.00
To avoid unplanned pregnancies	7	28.00
Infection control (avoid transmission of HIV)	3	12.00
Planned not to have any more children	3	12.00

### Factors associated with contraceptive uptake among women in the immediate postpartum period at Kawempe Hospital

Bivariate analysis was done using logistic regression to select the variables for inclusion in the logistic regression model. The variables with a *p* value less than 0.2 were selected for multivariate analysis. Among the socio-demographic characteristics, age was selected for further analysis as shown in Table 6. Among the obstetrics factors, these factors were selected for further analysis: parity, ever had an abortion, number of children alive, mode of delivery, planned time for next pregnancy, number of ANC visits attended, contraceptive counseling during, ANC, and method of contraceptive used before current birth (Table 7).

**Table 6:** Bivariate association of sociodemographic factors with contraceptive uptake among women in immediate postpartum period at Kawempe Hospital

Variable	Contraceptive uptake		cOR	95% CI	*p* value
	n(%)	95% CI for proportion			
Age					
18–24 years	22 (11.6)	7.8–17.1	1		
25–34 years	28 (17.6)	12.4–24.4	1.62	0.89–2.97	**0.116**
35 years and above	11 (22.4)	12.8–36.4	2.19	0.98–4.91	**0.055**
Marital status					
Married	52 (15.6)	12.1–19.9	1		
Single/divorced/separated/widow	9 (14.1)	7.4–25.0	0.88	0.41–1.90	0.752
Education level					
Primary/none	20 (16.1)	10.6–23.7	1		
Secondary	31 (14.0)	10.0–19.3	0.85	0.46–1.56	0.598
Tertiary	10 (19.2)	10.6–32.4	1.24	0.53––2.87	0.618
Occupation					
Housewife/unemployed	26 (14.7)	10.2–20.7	1		
Non professional	26 (16.1)	11.2–22.7	1.12	0.62 -2.02	0.710
Professional	9 (15.3)	8.1–27.0	1.05	0.46–2.38	0.916
Monthly household income (n = 390)					
10,000/= to 200,000/=	24 (14.5)	9.9–20.8	1		
200,001/= to 500,000/=	25 (17.5)	12.1–24.6	1.24	0.68–2.29	0.483
Above 500,000/=	9 (11.0)	5.8–19.9	0.72	0.32–1.64	0.439
Age of spouse/partner					
19–24 years	9 (18.0)	9.6–31.3	1		
25–34 years	25 (11.4)	7.8–16.3	0.58	0.25–1.34	0.206
35 years and above	26 (20.5)	14.4–28.6	1.18	0.51–2.75	0.693
Education level of spouse/partner					
None or N/A	5 (20.8)	8.8–41.9	1		
Primary	11 (14.7)	8.3–24.7	0.65	0.20–2.11	0.477
Secondary	36 (15.9)	11.6–21.3	0.72	0.25–2.04	0.532
Tertiary	9 (12.7)	6.7–22.7	0.55	0.16–1.85	0.334
Occupation of spouse/partner					
Non professional	44 (14.9)	11.2–19.4	1		
Professional	13 (15.5)	9.2–25.0	1.05	0.54–2.05	0.890
Unemployed or N/A	4 (23.5)	8.8–49.5	1.76	0.55–5.65	0.341
Spouse involvement in decision making					
No	15 (18.1)	11.1–27.9	1		
Yes	46 (14.6)	11.1–19.0	0.78	0.41–1.48	0.443

Bold values indicate variables with *p* value < 0.2

**Table 7:** Obstetric factors associated with contraceptive uptake at bivariate level among women in their immediate postpartum period at Kawempe Hospital

Variable	Contraceptive uptake		cOR	95% CI	*p* value
	n(%)	95% CI for proportion			
Parity					
Primipara/multipara (Less than 5)	48 (13.8)	10.5–17.8	1		
Grandmultipara (5 and above)	13 (27.1)	16.3–41.5	2.33	1.52 –4.72	**0.019**
Ever had an abortion					
No	40 (13.6)	10.1–18.0	1		
Yes	21 (20.4)	13.6–29.3	1.63	0.91–2.92	**0.103**
How many alive children					
One child	22 (13.8)	9.2–20.0	1		
Two to three children	20 (13.1)	8.6–19.4	0.94	0.49–1.81	0.860
Four or more children	19 (22.6)	14.9–32.9	1.83	0.93–3.62	**0.081**
Mode of delivery					
Vaginal delivery	28 (12.3)	8.6–17.3	1		
Caesarean delivery	33 (19.4)	14.1–26.1	1.71	0.99–2.96	**0.055**
Birth interval (n = 271)					
< = 24 months	14 (17.1)	10.3–26.9	1		
25–48 months	19 (18.6)	12.2–27.5	1.11	0.52–2.38	0.785
> 48 months	12 (13.8)	8.0–22.8	0.78	0.34–1.80	0.555
Planned time for next pregnancy					
None	17 (23.9)	15.4–35.3	1		
Within 1–2 years	4 (21.1)	7.9–45.4	1.27	0.35–4.58	0.714
More than 2 years	40 (13.0)	9.7–17.3	0.48	0.25–0.90	**0.023**
Number of ANC visits attended (n = 396)					
< = 3 visits	30 (20.1)	14.4–27.4	1		
> = 4 visits	31 (12.6)	9.0–17.3	0.57	0.33–0.97	**0.045**
Contraceptive counseling during ANC					
No	13 (7.7)	4.5–12.9	1		
Yes	48 (21.0)	16.1–26.7	3.16	1.65–6.05	**0.001**
Ever used contraception					
No	20 (12.7)	8.3–18.9	1		
Yes	41 (17.2)	12.9–22.5	1.42	0.80–2.54	0.226
Method used before (n = 238)					
Short acting reversible methods	34 (18.1)	13.1–24.3	1		
Long acting reversible methods	6 (12.0)	5.4–24.5	0.62	0.24–1.57	**0.310**

Bold values indicate variables with *p* value < 0.2

Multivariate logistic regression analysis was done to determine the factors that were independently associated with contraceptive uptake (Table 8). The factors that were significantly associated with contraceptive uptake with a *p* value less than 0.05 were; parity, mode of delivery, and contraceptive counseling during ANC. Grand multiparous women were 2.5 times more likely to take up contraception in the immediate postpartum period compared to Primiparous or multiparous women (aOR = 2.57; 95% CI 1.11–5.95; *p* = 0.028). Women who had delivered by cesarean section were 2.6 times more likely to take up contraception compared to women who had a vaginal delivery (aOR = 2.63; 95% CI 1.24–5.57; *p* = 0.011).

**Table 8:** Factors associated with contraceptive uptake at multivariate level among women in their immediate postpartum period at Kawempe Hospital

Variable	cOR (95% CI)	*p* value	aOR (95% CI)	*p* value
Parity				
Primipara/multipara (Less than 5)	1			
Grandmultipara (5 and above)	2.33 (1.52 –4.72)	0.019	2.57 (1.11–5.95)	0.028*
Mode of delivery				
Vaginal delivery	1			
Caesarean delivery	1.71 (0.99–2.96)	0.055	2.63 (1.24–5.57)	0.011*
Contraceptive counseling during ANC				
No	1			
Yes	3.16 (1.65–6.05)	0.001	9.05 (2.65–30.93)	< 0.001*
Method used before (n = 238)				
Injectable contraception (DMPA)	1			
Progestin only pill	0.43 (0.15–1.40)	**0.188**	0.50 (0.13–1.85)	0.299
Implants	0.77 (0.27–2.16)	0.615	0.64 (0.21–1.93)	0.428
IUD/condoms	1.23 (0.42–3.62)	0.701	1.18 (0.37–3.80)	0.779

Bold values indicate variables with *p* value < 0.05

Women who had received contraceptive counseling during antenatal care were 9 times more likely to take up contraceptive methods compared to women who had not (aOR = 9.05; 95% CI 2.65–30.93; *p* =  < 0.001).

The method of contraception used before was a confounder for the relationships between parity, mode of delivery, contraceptive counseling during ANC, and contraceptive uptake.

## Discussion

Within this study, the contraceptive uptake in the immediate postpartum period was 15.4%. This was comparatively lower than the 50.3% contraceptive uptake found at two district hospitals in Kenya [11]. The discrepancy in uptake can be explained by the difference in the delivery of contraceptive counseling. Women within the Kenyan hospitals received contraceptive counseling and there were staff members available to immediately offer the method selected by the women. However, the majority of women in this study were not aware of the possibility of obtaining contraception in the immediate postpartum period. In addition, there was about 42.3% of the women in this study who did not receive any form of contraceptive counseling.

The prevalence of contraceptive counseling in this study was also lower than 57% which was reported in a study done in Mexico among immediate postpartum women [12]. In Mexico,contraception is readily available for the mothers in the immediate postpartum period before discharge while in Kawempe Hospital the family planning clinic is far from the postnatal ward which increases the difficulty for the women to easily access and obtain the contraceptive methods before they are discharged. This shows that there is a need for postnatal wards to have staff members that are responsible for offering the family planning methods as well as contraceptive counseling for the women who missed it during the antenatal visits.

The contraceptive uptake within the immediate postpartum period is notably lower than that reported for women in the later postpartum period as shown by a study done within 12 states in the United States of America (USA) which reported a prevalence of 88.5% [13]. This can be explained by the fact that some women first want to heal before considering getting any method. Another reason for the low uptake highlighted for the women is that they want to discuss with their spouses the various contraceptive options. This period of decision-making impedes the uptake of contraception before discharge.

This study found that the progestin-only pills were the most commonly used method (40.4%) followed by implants (28.1%) and condoms (19.3%). This distribution greatly differs from that within the Mexican study where 38% of the women opted for IUDs and implants were selected by 1% only [12]. In Uganda, women have a negative attitude towards IUDs as shown in a study done by Twesigye et al. (2016) among 1,505 women in Ugandan public and private health facilities [14]. More than half of the women believed that the IUD reduces sexual pleasure, can cause damage to the womb, and can cause cancer as well.

The least selected method of contraception among the women in this study was Bilateral Tubal Ligation at 12.3%. The low percentage is explained by the fact that these women had delivered by C-section and opted to have the procedure done immediately after delivery of their babies which is similar to findings in a cohort study done in the USA [15]. This study showed that women who had vaginal deliveries usually requested tubal ligation 2–9 months postpartum. The financial status of individuals may also influence whether or not they choose tubal ligation since it is an operation that needs to be paid for in addition to the hospital delivery costs [15].

### Discussion of factors associated with contraceptive uptake

The factors associated with contraceptive uptake in this study include parity, mode of delivery, and contraceptive counseling during Antenatal care.

Grand multiparous women were 2.5 times more likely to take up contraception in the immediate postpartum period compared to Primiparous or multiparous women (aOR = 2.57; 95% CI 1.11–5.95; *p* = 0.028). This is very similar to findings in a population-based study done in Uganda which showed that women who had more children were more likely to adopt contraception in the immediate postpartum period since they wanted to limit the number of children compared to those who had fewer children [16]. According to our study, women who delivered by cesarean section were 2.6 times more likely to take up contraception compared to women who had a vaginal delivery (aOR = 2.63; 95% CI 1.24–5.57; *p* = 0.011). Similar findings were reported in the Mexican study [12]whereby women who delivered by urgent cesarean had a 56% more likelihood of taking up a contraceptive method compared to women who had a vaginal delivery. Usually, women who deliver by cesarean section have higher utilization of health facilities during ANC (for those with elective cesarean procedures) than those who deliver vaginally. These women delivering by C/S also highly utilize health facilities during delivery since they stay admitted for a longer time compared to those who undergo vaginal delivery. High utilization of critical services has been shown to encourage subsequent contraceptive use and reduce the unmet need for family planning [17]. Since there is a need for healing of the scar, women who have been delivered by C/S are encouraged to space their pregnancies or to limit their children after about 4 C/S. this in turn leads to increased uptake of immediate postpartum contraception, especially through permanent methods since tubal ligation can be performed during the procedure [17].

According to our study, women who had received contraceptive counseling during antenatal care were 9 times more likely to take up contraceptives in the immediate postpartum period (aOR = 9.05; 95% CI 2.65–30.93; *p* =  < 0.001). This was in agreement with a study done in Mexico [12] and in Uttar Pradesh (India) [17]. Counseling on family planning leads to an increased likelihood to use contraception [18] however in most developing countries where accessing functional facilities is challenging many women are unable to return for their 6-week postpartum visits and thus unable to receive FP counseling and adopt a method [19]. Therefore ensuring that the mothers receive counseling during ANC enables them to make an informed decision so that they can adopt a method of contraception immediately after delivery even before being discharged.

## Conclusions and recommendations

The contraceptive uptake among immediate postpartum women was 15.4% which is very low. The factors associated with contraceptive uptake among immediate postpartum mothers were parity, mode of delivery, and contraceptive counseling during Antenatal care. In light of these findings, we recommend the following: Counseling of women should be done during ANC and after delivery to inform them about the immediate postpartum contraception and how it is done. This counseling should also include information on the importance of applying birth interval or appropriate child spacing. Community sensitization should be carried out to increase awareness about immediate postpartum contraception. The ministry of health sector of government should create policies regarding immediate postpartum contraception among women. There is also a need for further research to assess contraceptive uptake after the implementation of antenatal counseling. Research can also be done to assess the effect of immediate postpartum contraception on the  prevention of unplanned pregnancies and associated consequences including abortions, short birth intervals, and perinatal mortality.

### Limitations

Generalizability is a problem since the study only involved women delivering at a tertiary center where health providers with skills in postpartum contraception were available. Results may not be able to apply to women in the rural setting because of the difference in societal and contextual factors. Since there were inadequate numbers, certain variables were unable to come out as significant for example Contraceptive methods used before.

## Data Availability

The datasets used and/or analyzed during the current study are available from the corresponding author on reasonable request.

## Abbreviations

BTL: Bilateral Tubal ligation
DHS: Demographic Health Survey
IPPF: International Planned Parenthood Federation
IUD: Intra Uterine Device
LARC: Long Acting Reversible Contraceptives
MAKCHS: Makerere University College of Health Sciences
MMR: Maternal Mortality Ratio
PI: Principal Investigator
PPFP: Post-Partum Family Planning
RA: Research Assistant
TFR: Total Fertility Rate
UBOS: Uganda Bureau of Statistics
UDHS: Uganda Demographic Health Survey
UN: United Nations
UNFP: United Nations Population Fund
USAID: United States Agency for International Development
WHO: World Health Organization

## References

[1] UN (2015). Trend in contraceptive use worldwide 2015.

[2] WHO. Postpartum family planning: essential for ensuring health of women and their babies. 2018. Cited 18 Sept 2019. https://www.who.int/reproductivehealth/topics/family_planning/world-contraception-day-2018/en/.

[3] UBOS and ICF. Uganda demographic and health survey 2016: key indicators report. 2017. Kampala, Uganda: UBOS, and Rockville, Maryland, USA: UBOS and ICF.

[4] Thwaites A (2018). Immediate postnatal contraception: what women know and think. BMJ Sex Reprod Health.

[5] Bakamjian L. Programming strategies for postpartum family planning. 2013.

[6] WHO (2015). Medical eligibility criteria for contraceptive use.

[7] Cleland J (2012). Contraception and health. Lancet.

[8] Achyut P (2016). Integration of family planning with maternal health services: an opportunity to increase postpartum modern contraceptive use in urban Uttar Pradesh, India. J Fam Plan Reprod Health Care.

[9] Kozuki N, Lee AC, Silveira MF (2013). The associations of parity and maternal age with small-for-gestational-age, preterm, and neonatal nd infant mortality: a meta-analysis. BMC Public Health.

[10] Moniz M (2017). Immediate postpartum long-acting reversible contraception: the time is now. Contraception.

[11] Shabiby MM (2015). Factors influencing uptake of contraceptive implants in the immediate postpartum period among HIV infected and uninfected women at two Kenyan District Hospitals. BMC Womens Health.

[12] Darney B (2016). The relationship of age and place of delivery with post-partum contraception prior to discharge in Mexico: a retrospective cohort study. Contraception.

[13] Whiteman M (2009). Contraceptive use among postpartum women-12 states and New York City, 2004–2006. Morb Mortal Wkly Rep.

[14] Twesigye R (2016). Ugandan women's view of the IUD: generally favorable but many have misperceptions about health risks. Glob Health Sci Pract.

[15] Thurman AR, Janecek T (2010). One-year follow-up of women with unfulfilled postpartum sterilization requests. Obstet Gynecol.

[16] Rutaremwa G (2015). Predictors of modern contraceptive use during the postpartum period among women in Uganda: a population-based cross sectional study. BMC Public Health.

[17] Yadav D, Dhillon P (2015). Assessing the impact of family planning advice on unmet need and contraceptive use among currently married women in Uttar Pradesh, India. PLoS ONE.

[18] Do M, Hotchkiss D (2013). Relationships between antenatal and postnatal care and post-partum modern contraceptive use: evidence from population surveys in Kenya and Zambia. BMC Health Serv Res.

[19] Gallagher MC (2021). Immediate postpartum long-acting reversible contraception: a comparison across six humanitarian country contexts. Front Glob Womens Health.

